# Intrarater and interrater reliability of first metatarsophalangeal joint dorsiflexion: goniometry versus visual estimation

**DOI:** 10.1186/1757-1146-3-S1-P5

**Published:** 2010-12-20

**Authors:** Sarah Curran, Angela Jones

**Affiliations:** 1 University of Wales Institute, Cardiff, Cardiff, UK

## 

Visual estimation (VE) and goniometry measurements (GM) are commonly used to assess first metatarsophalangeal joint (MTPJt) dorsiflexion. The purpose of this study was to determine the intra and interrater reliability of VE and GM and to establish if reliability was influenced by the experience of an examiner. On two separate occasions, ten experienced and ten inexperienced examiners evaluated three clinical, real size photographs. Experienced examiners demonstrated excellent intra and interrater reliability for GM (Intraclass correlation coefficient [ICC] >0.953; standard error of measurement [SEM] 1.8Â° – 2.5Â°), compared to inexperienced examiners who showed fair-to-good intra and interrater reliability (ICC 0.322 - 0.597; SEM, 2Â° - 3Â°). For VE, inexperienced examiners demonstrated fair-to-good interrater and excellent intrarater reliability (ICC 0.666 – 0.808), and was higher compared to the experienced examiners (ICC 0.167 – 0.672). The SEM (2.8Â°- 4.4Â°) was less varied than that of experienced examiners (SEM 3.8Â° – 6.4Â°) for VE, but neither group’s SEM were clinically acceptable. Whilst minimal differences between intra and interrater reliability of GM and VE are noted, the study suggests that GM is more reliable than VE when used by experienced examiners. These findings support continued use of GM for first MTPJt dorsiflexion assessment.

